# Primary *Actinomyces* in Hand: An Unusual Cause of Osteomyelitis

**DOI:** 10.1002/ccr3.70298

**Published:** 2025-03-04

**Authors:** Saman Al‐Zahawi, Alireza Ghanadan, Alireza Hadizadeh, Faeze Salahshour, Ali Reza Khosravi, Zahra Saffarian

**Affiliations:** ^1^ Department of Dermatology Razi Hospital, Tehran University of Medical Sciences Tehran Iran; ^2^ Department of Dermatopathology Razi Hospital, Tehran University of Medical Sciences (TUMS) Tehran Iran; ^3^ Female Pelvic Medicine and Reconstructive Surgery, Division of Urogynecology University of Chicago, Northshore University Health System Chicago Illinois USA; ^4^ Department of Radiology, Advanced Diagnostic and Interventional Radiology Research Center Tehran University of Medical Sciences Tehran Iran; ^5^ Faculty of Veterinary Medicine University of Tehran Tehran Iran; ^6^ Department of Dermatology Imam Khomeini Hospital Complex, Tehran University of Medical Sciences Tehran Iran

**Keywords:** actinomycosis, antibiotics, ceftriaxone, hand, osteomyelitis, trimethoprim sulfamethoxazole

## Abstract

Actinomycosis is a rare, chronic, and slow‐progressing bacterial infection caused by *Actinomyces species*. The condition's rarity, nonspecific clinical symptoms, and occasional occurrence in atypical locations often lead to delays in diagnosis, which are critical for initiating timely treatment and preventing further complications. We report the case of an 82‐year‐old man who initially developed pustules on the dorsum of his left hand, which gradually progressed over 15 years into an exophytic mass. Histopathological examination and culture confirmed Actinomycosis, and imaging studies revealed osteomyelitis affecting the carpal and metacarpal bones of the involved hand. Although the delayed diagnosis and treatment resulted in severe osteomyelitis, the patient responded favorably to a combination therapy of Amoxicillin and Trimethoprim‐Sulfamethoxazole.

**Summary**

- Hand actinomycosis can present with skin lesions that mimic various other dermatological conditions, such as squamous cell carcinoma, mycobacterial infections, sporotrichosis, and leishmaniasis.
- This similarity can make diagnosis challenging, as these conditions require different treatments.

## 1 Introduction

*Actinomycetes* are a group of gram‐positive anaerobes that were previously classified as fungi. These bacteria normally colonize the mouth, colon, and vagina. *Actinomyces israelii* is the most common pathogenic *Actinomyces* in humans [1]. Cervicofacial lesions account for two‐thirds of cases of actinomycosis, followed by abdomino‐pelvic and thoracic lesions. In rare instances, the primary infection may occur in the hands with extension of the lesion to the underlying bone, resulting in severe osteomyelitis and a possible risk of amputation due to delay in diagnosis [2]. The clinical presentation typically includes a suppurative plaque, fibrosis, granulomatous inflammation, and the formation of sinus tracts [3]. The initial suppurative plaque stage may be confused with cutaneous tuberculosis or other atypical mycobacterial infections, and the sinus tract in the Cervicofacial subtype may mimic scrofuloderma, but the typical finding of Sulfur granules in biopsy will aid in distinguishing this rare clinical entity from the aforementioned conditions. Patients with mass lesions who are unable to undergo surgical debridement may opt for prolonged antibiotic therapy [4]. The preferred antibiotic drug for actinomycosis is Penicillin G or ampicillin [1]. *Actinomycetes* are also sensitive to a broad range of antibiotic drugs including sulfonamides, erythromycin, doxycycline, and tetracycline [5]. When lesions do not respond to antibiotic therapy, surgical excision is considered a final, fundamental treatment option [6].

## 2 Case History/Examination

An 82‐year‐old man presented with an exophytic mass on the back of his left hand. This lesion began 15 years prior as a few pustules on a red base. Over time, these pustules enlarged, coalescing into a suppurative plaque. Five years before his presentation at our center, a surgeon, suspecting an abscess, attempted drainage. This procedure, along with multiple courses of intravenous ceftriaxone, was followed by further growth of the lesion. The suppurative plaque continued to expand, eventually transforming into the exophytic mass, which by the time he came to us had spread to the palm of his hand, forming a verrucous plaque there. The patient was immunocompetent, reported no cough or other respiratory symptoms, and his only medication was Prazocin for benign prostatic hyperplasia.

### 2.1 Differential Diagnosis, Investigations and Treatment

**FIGURE 1:**
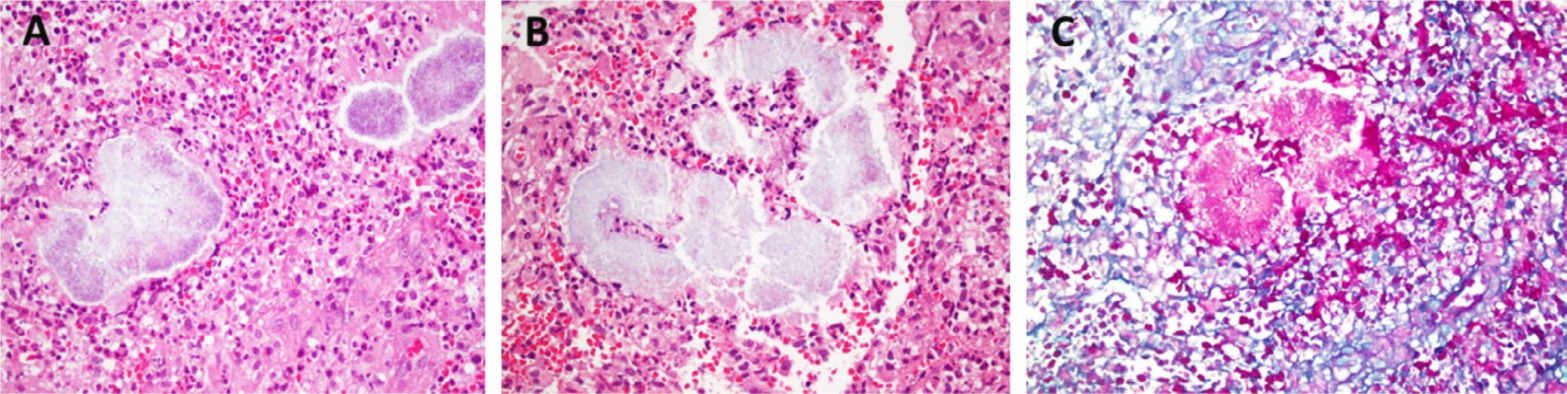
Histopathologic examination of an ellipse biopsy from the dorsum of the hand (A) High power of the light grain shows filamentous organism surrounded by mainly neutrophils and epithelioid histiocytes as well as some lymphocytes (H&E X 100 original magnification). (B) In this plane, predominant neutrophils are surrounding light grains (H&E X 100 original magnification). (C) PAS stain demonstrates filamentous organism in between inflammatory cell infiltrate (X 100 original magnification).

Five years after the initial presentation, a biopsy was performed, but it was inconclusive, showing only a granulomatous reaction. While subsequent biopsies raised the suspicion of actinomycosis, the diagnosis was finally suggested by a biopsy at our center (Figure 1A–C). To confirm the possibility of actinomycotic mycetoma and to rule out atypical mycobacterial infection, a culture was performed. This culture revealed thin, branching filaments, consistent with actinomycotic mycetoma. It is important to remember that actinomycosis can resemble other conditions such as tuberculosis, fungal infections, botryomycosis, and malignancy [7].

**FIGURE 2:**
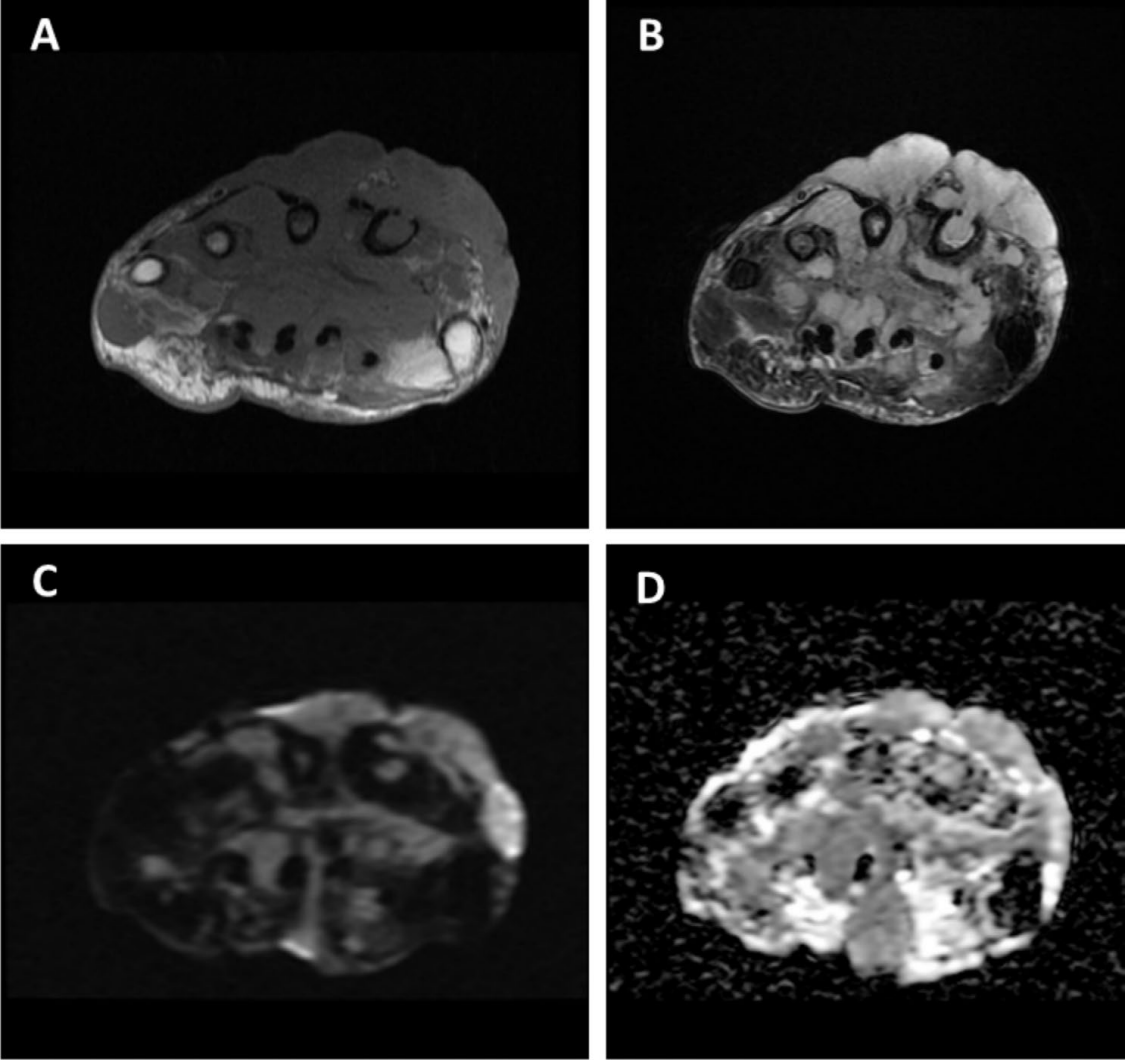
The Axial T1 and T2 (A, B) weighted images showed abnormal signals indicative of osteomyelitis in the distal row of carpal bones and proximal regions of the second, third, and fourth metacarpals. Additionally, cortical destruction of the second metacarpal bone was observed. The MRI images also demonstrated the presence of collections in both the volar and dorsal sides of the hand, extending into the intercarpal spaces. The volar abscess had compressed and displaced the carpal tunnel volarly (C). (D) ADC map.

The patient also reported paresthesia, hand pain, and wrist pain in the affected hand, prompting an X‐ray, which revealed signs of osteomyelitis. MRI was then performed to further evaluate the extent of the infection and confirm the osteomyelitis diagnosis (Figure 2A–D).

The patient's diagnosis was determined to be actinomycotic mycetoma based on biopsy, culture, and imaging findings. The MRI's abnormal signals, along with cortical bone destruction in the metacarpal and the presence of abscesses in the hand, confirm the diagnosis of osteomyelitis.

### 2.2 Outcome and Follow Up

**FIGURE 3:**
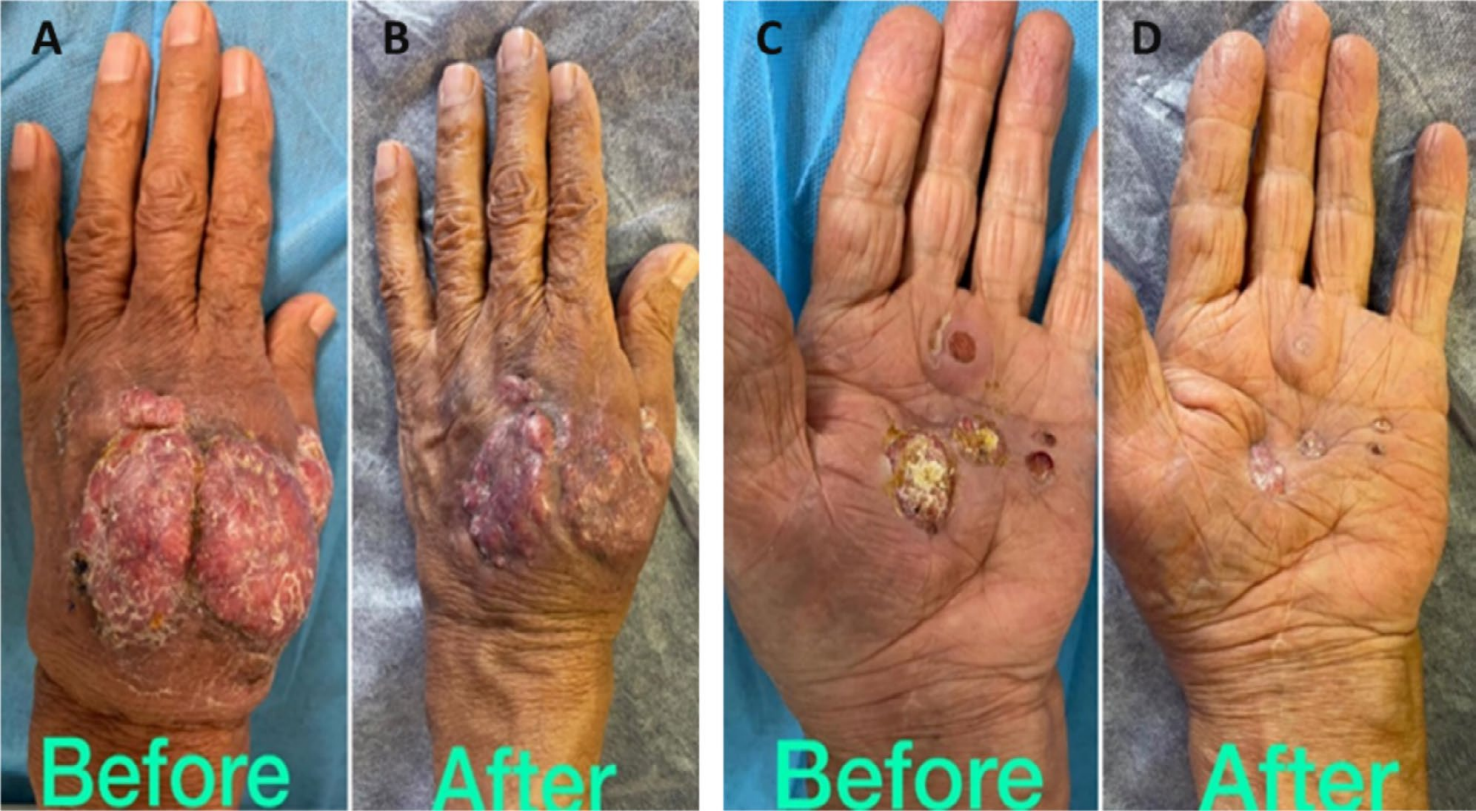
Dorsum of the hand showing exophytic mass before treatment and significant resolution after treatment (A, B), also the verrucous nodules of the palm have been resolved after treatment with slight skin retraction (C, D).

Due to the severity of the condition, an orthopedic consultant recommended extensive debridement, potentially including amputation. However, the patient declined surgical intervention. Consequently, an infectious disease specialist initiated antibiotic therapy with amoxicillin and trimethoprim‐sulfamethoxazole. Fortunately, the patient responded positively to this treatment, with the lesion shrinking, reduced vascular engorgement, and resolution of the paresthesia (Figure 3A–D).

## 3 Discussion

This patient's case presents a complex diagnostic challenge. The initial pustules on the back of his left hand progressed and recurred despite multiple courses of short‐term antibiotic treatment. Histopathological evaluation eventually confirmed a delayed diagnosis of actinomycosis, and imaging studies revealed osteomyelitis in the carpal bones and proximal metacarpals, along with multiple fluid collections and joint destruction. Delayed diagnosis of actinomycosis is common, likely due to its nonspecific clinical presentation, subacute/chronic nature, often negative culture results, the absence of pathognomonic sulfur granules in some cases, and, as in this instance, atypical presentation in locations distant from the bacteria's usual habitat in the mouth and genitalia. It is noteworthy to mention that only 24% of cases of actinomycosis are culture positive [8]. Delayed diagnosis of actinomycosis can lead to various complications depending on the primary infection site. For example, hand actinomycosis can result in osteomyelitis of the underlying bone, while disseminated actinomycosis originating from a primary pulmonary infection can cause meningitis or endocarditis [9] Actinomycosis should be suspected when lesions are preceded by trauma, occur in immunocompromised individuals, or are long‐standing, suppurative lesions with draining fistulae.

Although osteomyelitis of the vertebral column and jaw bone from Cervicofacial actinomycosis is more frequently reported, primary actinomycosis of the hand with bone involvement is very rare; only a few cases have reported osteomyelitis secondary to primary actinomycosis of the hand due to direct extension of the infection to the underlying bone [10, 11]. Additionally, Actinomycotic osteomyelitis may occur in the setting of immunosuppression by hematogenous spread [12].

Treatment of the initial pustular or early erythematous plaque may be achieved by long‐term antibiotic monotherapy and frequent follow‐up for complete resolution. Late lesions such as sinus tract, suppurative plaque, and mass lesions are treated by a combination of surgical debridement and long‐term appropriate antibiotic therapy. Also, treatment of late lesions of actinomycosis with only antibiotics is possible in patients refusing surgical debridement or having contraindications to surgical operation [13].

Lastly, it is crucial to closely monitor the patient's progress and continue the antibiotic therapy to ensure complete remission and prevent any further complications. Regular follow‐up examinations and imaging studies will help assess the effectiveness of the treatment and guide further management decisions.

## 4 Conclusion

Cutaneous actinomycosis is frequently misdiagnosed due to its rarity, varied and nonspecific clinical presentations, and occasional atypical locations on the extremities. Untreated, long‐standing lesions in these areas can spread to adjacent tissues, such as bone, leading to serious complications like osteomyelitis. Early clinical diagnosis and prompt treatment are crucial for minimizing such complications.

## Author Contributions

**Saman Al‐Zahawi:** writing – original draft, writing – review and editing. **Alireza Ghanadan:** visualization. **Alireza Hadizadeh:** writing – review and editing. **Faeze Salahshour:** data curation, visualization. **Ali Reza Khosravi:** supervision. **Zahra Saffarian:** conceptualization, supervision.

## Consent

Written informed consent was obtained from the patient to publish this study in accordance with the journal's patient consent policy.

## Conflicts of Interest

The authors declare no conflicts of interest.

## Data Availability

The authors elect to share data upon request.

## References

[1] Y. Han , Y. Cao , Y. Zhang , L. Niu , S. Wang , and C. Sang , “A Case Report of Pelvic Actinomycosis and A Literature Review,” American Journal of Case Reports 21 (2020): e922601‐1. 32532952 10.12659/AJCR.922601 PMC7476745

[2] S. Padhi , M. Dash , J. Turuk , R. Sahu , and P. Panda , “Primary Actinomycosis of Hand,” Advanced Biomedical Research 3, no. 1 (2014): 225. 25538911 10.4103/2277-9175.145700 PMC4260285

[3] J. Garner , M. Macdonald , and P. Kumar , “Abdominal Actinomycosis,” International Journal of Surgery 5, no. 6 (2007): 441–448. 18078685 10.1016/j.ijsu.2006.06.009

[4] K. Moturi and V. Kaila , “Cervicofacial Actinomycosis and Its Management,” Annals of Maxillofacial Surgery 8, no. 2 (2018): 361–364. 30693266 10.4103/ams.ams_176_18 PMC6327805

[5] F. Valour , A. Sénéchal , C. Dupieux , et al., “Actinomycosis: Etiology, Clinical Features, Diagnosis, Treatment, and Management,” Infection and Drug Resistance 7 (2014): 183–197. 25045274 10.2147/IDR.S39601 PMC4094581

[6] O. Oostman and R. A. Smego, Jr. , “Cervicofacial Actinomycosis: Diagnosis and Management,” Current Infectious Disease Reports 7, no. 3 (2005): 170–174. 15847718 10.1007/s11908-005-0030-0

[7] V. Indushekar , I. L. Jeswani , S. Goyal , M. Punjabi , and C. B. Patil , “Primary Cutaneous Actinomycosis of Scrotal Skin: A Rare Entity Often Misdiagnosed,” Indian Journal of Pathology and Microbiology 59, no. 2 (2016): 243–244. 27166055 10.4103/0377-4929.174852

[8] N. Golden , H. Cohen , J. Weissbrot , and S. Silverman , “Thoracic Actinomycosis in Childhood,” Clinical Pediatrics 24, no. 11 (1985): 646–650. 4053481 10.1177/000992288502401113

[9] S. Sharma , M. F. Hashmi , and D. J. Valentino, III , Actinomycosis (StatPearls, 2018).

[10] G. Rushforth and S. J. Eykyn , “Actinomycosis of the Hand,” Hand 14, no. 2 (1982): 194–197. 7117937 10.1016/s0072-968x(82)80017-x

[11] R. J. Blinkhorn , “Punch'actinomycosis Causing Osteomyelitis of the Hand,” Archives of Internal Medicine 148, no. 12 (1988): 2668–2670, 10.1001/archinte.1988.00380120112022. 3058075

[12] D. J. Ryu , Y. S. Jeon , H. Y. Kwon , S. J. Choi , T. H. Roh , and M. K. Kim , “Actinomycotic Osteomyelitis of a Long Bone in an Immunocompetent Adult: A Case Report and Literature Review,” BMC Musculoskeletal Disorders 20, no. 1 (2019): 1–8, 10.1186/s12891-019-2576-2. 31043170 PMC6495508

[13] S. S. Sethy , V. Singh , A. K. Choudhury , G. Singh , P. K. Gupta , and V. Mehta , “Actinomycotic Osteomyelitis of the Hand and Wrist Treated With Pharmacotherapy Alone: A Case Report,” JBJS Case Connector 10, no. 3 (2020): e19, 10.2106/JBJS.CC.19.00520. 32668139

